# HIV related pulmonary arterial hypertension: epidemiology in Africa, physiopathology, and role of antiretroviral treatment

**DOI:** 10.1186/s12981-015-0078-3

**Published:** 2015-11-11

**Authors:** Jean Joel R. Bigna, Paule Sandra D. Sime, Sinata Koulla-Shiro

**Affiliations:** Department of Epidemiology and Public Health, Pasteur Center of Cameroon Member of International Network of the Pasteur Institutes, Yaoundé, Cameroon; Faculty of Medicine and Biomedical Sciences, University of Yaoundé 1, Yaoundé, Cameroon; Infectious Diseases Unit, Yaoundé Central Hospital, Yaoundé, Cameroon

**Keywords:** HIV, AIDS, Pulmonary hypertension, Pulmonary arterial hypertension, Africa, Antiretroviral therapy

## Abstract

The development of HIV related pulmonary arterial hypertension (PAH) reduces the probability of survival by half as compared with HIV-infected individuals without HIV related PAH. HIV infected patients have a greater incidence of PAH compared to general population and have a 2500-fold increased risk of developing PAH. It is therefore important to have a recent overview of the problem in Africa, the most HIV affected part of the world (70 % of all HIV infection in the world). First, we discussed the epidemiology of HIV-related PAH in Africa. Second, the current understanding of the HIV-related PAH pathogenesis has been covered. Third, role of highly active antiretroviral therapy on HIV-related PAH has been revisited. There are few data concerning epidemiology of HIV related pulmonary hypertension in Africa leading to necessity to conduct further prospective large studies. The prevalence of PAH among HIV infected people in Africa varies from 5 to 13 %. The prevalence of HIV-related PAH in Africa is notably high compared to those in developed countries and in general population. The pathogenesis of PAH is clearly complex, and probably results from the interaction of multiple modulating genes with environmental factors. The physiopathology includes cytokines secretion increase which induces dysregulation of endothelial and vascular smooth muscle cell growth and imbalance of endogenous vasodilators and constrictors; HIV viral proteins which induces vascular oxidative stress, smooth myocyte proliferation and migration, and endothelial injury and genetic predisposition due to some major histocompatibility complex alleles, particularly HDL-DR6 and HLA-DR5. Histologically, HIV related PAH has the same characteristics with other types PAH. Antiretroviral therapy have a beneficial effect on the outcome of HIV related pulmonary hypertension, but it lacks evidence from large prospective studies.

## Background

Pulmonary hypertension (PH) is defined as mean pulmonary arterial pressure (mPAP) ≥25 mmHg on right heart catheterization at rest [1]. The classification of PH involves five groups: pulmonary arterial hypertension (PAH) which includes human immunodeficiency virus (HIV) related PH, PH due to left heart diseases, PH due to respiratory diseases and/or hypoxemia, PH due to chronic embolic disease, and PH having unclear multifactorial mechanism [1]. PAH is defined by a mPAP ≥25 mmHg, pulmonary capillary wedge pressure ≤15 mmHg and pulmonary vascular resistance >3 Wood units at the time of right heart catheterization [1].

Symptoms result from right ventricular dysfunction. The first clinical manifestation is effort intolerance and exertional dyspnea that will progress to the point of breathlessness at rest. These symptoms are nonspecific, of course, and overlap with those of many other diseases, including pulmonary and cardiac conditions. Echocardiography is an extremely useful tool for the diagnosis of HIV related PAH, and Doppler echocardiography can be used to estimate systolic pulmonary artery pressure. Assessment of hemodynamic measures by catheterization remains, however, the best test for evaluating the response to therapy. Cardiac catheterization is mandatory to definitively diagnose the disease and exclude any underlying cardiac shunt as the etiology [2].

About two-thirds of the deaths in patients with HIV-related PAH were due to the consequences of PAH, such as right heart failure, cardiogenic shock, and sudden death [3]. HIV infected patients have a greater incidence of PH compared to general population [4]. In comparison with the incidence of idiopathic PAH in the general population (1–2 per million), HIV-infected patients have a 2500-fold increased risk of developing PAH [5]. The development of HIV related PAH reduces the probability of survival by half as compared with HIV infected individuals without HIV related PAH. It therefore is important to have a recent overview of the problem in sub-Saharan Africa, the most HIV affected part of the world with 24.7 million people living with HIV (70 % of all the world) [6], and to summarize current knowledge on HIV-related PAH.

First, we discussed the epidemiology of HIV-related PAH in Africa. Second, the current understanding of the HIV-related PAH pathogenesis has been covered. Third, role of highly active antiretroviral therapy on HIV-related PAH has been revisited.

## Epidemiology of HIV related pulmonary arterial hypertension in Africa

There are few data concerning epidemiology of HIV related pulmonary hypertension in Africa which come all from cross-sectional studies (Table 1). A study conducted in 2012 including 116 vertically HIV-infected adolescents (10–19 years) in Zimbabwe revealed a prevalence of 7 % for PAH [7]. In another study including 102 HIV infected patients in Tanzania, pulmonary hypertension was present among 13 % of patients [8]. In a retrospective cross-sectional study conducted in Burkina-Faso among 79 HIV-infected patients, the prevalence was 5 % [9]. In a cross sectional study in South Africa including 518 HIV-infected patients, the prevalence was 8 % [10].

**Table 1:** Characteristics of studies reporting epidemiology of pulmonary hypertension among HIV infected people in Africa

First author name, Publication year	Study design	Patients characteristics	Prevalence	Diagnostic	Setting	Factors associated with PH
Ferrand, 2012 [7]	Cross sectional	N = 116 Mean age = 14 years (10–19) Women = 69 %	7 %	Mean pulmonary artery pressure >25 mmHg with Doppler echocardiography	Zimbabwe	Not researched
Chillo, 2012 [8]	Cross sectional	N = 102 Mean age = 42 years (18–72) Women = 69 %	Overall: 13 % Men: 19 % (6/32) Women: 10 % (7/70)	Pulmonary arterial pressure >35 mmHg with Doppler echocardiography	Tanzania	None (researched: SBP, DBP, duration of HIV infection, cholesterol level, age, gender and use of HAART)
Sliwa, 2012 [10]	Cross sectional	N = 518 Mean age = 42 years (18–72) Women = 62 %	Overall: 8 % Men : 6 % (11/197) Women: 10 % (31/321)	Pulmonary artery pressure >25 mmHg with Doppler echocardiography	South Africa	Not researched
Niakara, 2002 [9]	Retrospective cross sectional	N = 79 Mean age = 46 years Women = 44 %	5 %	Echocardiography	Burkina Faso	Not researched

*SBP* systolic blood pressure, *DBP* diastolic blood pressure, *HAART* highly active antiretroviral treatment

The prevalence of PH among HIV infected people in Africa varies from 5 to 13 %. With around 24.7 million of HIV infected people in sub-Saharan Africa [6], between 1.2 and 3.2 million might have PAH. There is no difference between the prevalence in male (6–19 %) and the prevalence in female (around 10 %). The prevalence in sub-Saharan Africa is notably high compared to those in developed countries where the prevalence is close to 0.5 % [4, 11–13]. The possible explanation is that, the diagnosis and the management of HIV infection were made at an advanced stage of HIV disease. And also, in most developed countries, antiretroviral therapy is initiated regardless CD4 count and HIV infection clinical stage compared to developing countries which take in account these parameters.

There is need for large prospective studies to better estimate burden of HIV related PAH in Africa. In all of the studies mentioned above, the diagnostic tool was echocardiography, therefore there is need studies using cardiac catheterization which gold standard [2] to better estimate epidemiology. As demonstrated by a study, echocardiographic assessment of pulmonary arterial pressure was inaccurate in 19.7 % of patients compared to right heart catheterization [14].

## Physiopathology of HIV related pulmonary hypertension

**Fig. 1:**
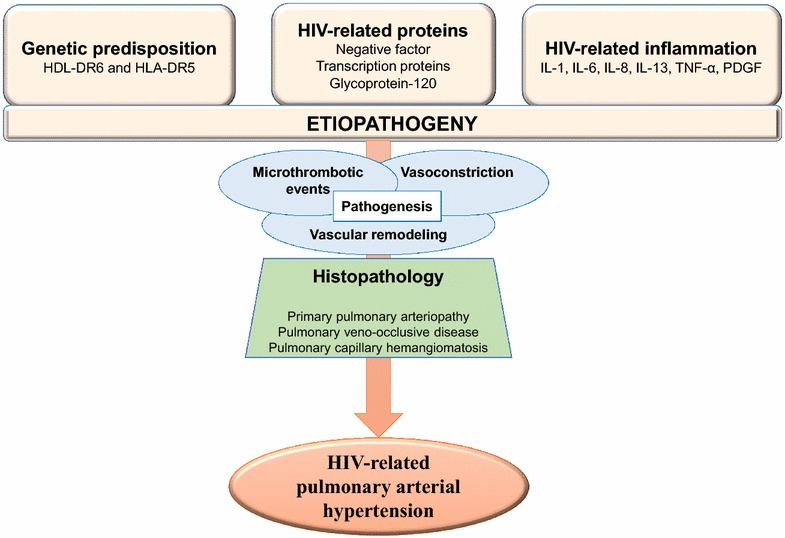
Physiopathology of HIV-related pulmonary hypertension

The pathogenesis of PAH is clearly complex, and probably results from the interaction of multiple modulating genes with environmental factors. The mechanism is unclear and to date not completely understood. Several key aspects involved in the pathophysiological process of HIV related PAH. HIV infection itself plays a major role in the development of PAH. The mechanism is not directly due to the action of the virus because attempts to localize the virus in the vascular lesions or endothelial cells of affected patients have been unsuccessful [15], suggesting that a direct role of the virus is unlikely, and indicating that the underlying mechanism in pulmonary arterial hypertension associated with HIV is related to the indirect action of infection, possibly through the action of viral proteins and chronic inflammation cytokines mediated due to HIV infection. Three main mechanisms are responsible for the HIV PAH: the HIV viral proteins found in the pulmonary vascular endothelium, cytokines due to the presence of HIV and increase the genetic predisposition due to HIV (Fig. 1). The pathogenesis of PAH is characterized by three major processes including vasoconstriction, vascular remodeling and microthrombotic events [16].

### Role of cytokines and inflammation

HIV infection induces high secretion of some cytokines by monocytes, macrophages and lymphocytes including interleukin (IL)-1, IL-6, IL8, IL-13, tumor necrosis factor (TNF) α and platelet-derived growth factors which can exacerbates a patent PAH or induces inflammation of vascular endothelium leading to PAH [16–23]. The activation of platelet derived growth factor [24] and vascular endothelial pathway [25] can result to aberrant pulmonary vascular activity. These growth factors and cytokines can lead to dysregulation of endothelial and vascular smooth muscle cell growth and imbalance of endogenous vasodilators and constrictors (in favor of constrictors). IL-1 appears to have deleterious effects for the development and progression of pulmonary hypertension. The exact mechanisms, however, remain unclear [16]. It was shown that elevated levels of IL-6 resulted in an upregulation of vascular endothelial growth factor receptor II and matrix metalloproteinase-9, an endopeptidase that promotes angiogenesis through regulation of cell attachment, proliferation, and migration [16, 26]. IL-8 is thought to play an important role in the development of PAH, especially in early phases of vascular remodeling. IL-8 is known to have proangiogenic and antiapoptotic activities and acts as a growth factor for endothelial cells [27]. IL-13 acts as an important mediator of cell proliferation and tissue remodeling in lungs [28]. This is explain by imbalance of NO homeostasis and increased muscularization of pulmonary arteries [16]. Similarly to other inflammatory cytokines, elevated serum levels of TNF-α were described in PAH patients. TNF-α might play an important role in the development of pulmonary hypertension, even though the concrete mechanisms remain unknown [29]. Interestingly some studies show that TNF-α blockers ameliorate pulmonary pressure, while other studies found no significant effects [16].

### Role of HIV viral proteins

These HIV viral proteins include negative factor (Nef), transcription proteins (Tat) and glycoprotein 120 (gp-120) [30–35]. These proteins probably induce vascular oxidative stress, smooth myocyte proliferation and migration, and endothelial injury leading to HIV related PAH.

Nef induces complex plexiform lesion in the pulmonary vasculature, this has been demonstrated in a study comparing macaque Nef-positive simian immunodeficiency virus (SIV) and macaque Nef-negative SIV [30]. The involvement of Nef in the occurrence of HIV-related PAH has been also demonstrated in porcine model study and among HIV infected persons [31, 33], because Nef is present in the endothelial cells of HIV infected patients with PAH. Nef can enter into the pulmonary endothelial cells via the CXCR 4 receptor and therefore induce proliferation and apoptosis of endothelial cells in lung [36, 37]. Thus, localization of Nef to the lipid rafts may be sufficient to trigger the changes associated with the endothelial cell expansion characteristic of plexiform lesions [38].

Bone morphogenetic protein type II receptor (BMPR2) participate in a multiplicity of ways in the regulation of numerous physiological and pathological processes including the inhibition of the proliferation of vascular smooth muscle tissue by promoting the survival of pulmonary arterial endothelial cells, therefore preventing arterial damage and adverse inflammatory responses [39]. Its wide-ranging biological functions is controlled by several mechanisms, including regulation of transcription, complex formation among the signaling receptors (oligomerization) and with co-receptors, binding of the receptors to scaffolding proteins or their targeting to specific membrane domains [39]. This control can be breakdown by a mutation the gene encoding to the BMPR2. A heterozygous germline mutations in the gene encoding the BMPR2), can lead to heritable PAH in more than 70 % of cases [40–42]. Tat represses transcription of gene encoding to BMPR2 in monocytes, suggesting that Tat-mediated reduction of BMPR2 may be linked to the development of HIV related PAH [43]. The BMPR2 is a member of the transforming growth factor-beta receptor family and is expressed on the surface of several cell types including endothelial cells and macrophages. The modulation of host gene transcription by HIV infection might genetically alter expression of BMPR2. As the HIV Tat protein is the major transcriptional regulator of host gene expression during HIV infection, Tat down-regulates BMPR2 expression and signaling by half. This level of decreased BMPR2 expression can lead to abnormal pulmonary vascular function with exuberant cellular proliferation [43]. Tat enhance the activity of vascular endothelial cells via interleukin-6 [44].

Secreted HIV gp120 proteins induce lung endothelial cell injury and could contribute to the development of HIV-related PAH. The mechanism is by apoptosis and smooth cell proliferation due to exposure of HIV-1 gp120 proteins to primary human lung microvascular endothelial cells [32]. Gp120 also significantly increases secretion of the potent vasoconstrictor endothelin-1 by human lung endothelial cells [45].

Nevertheless, it is essential to state that only a small proportion of infected patients with HIV will develop HIV related PAH. Other factors, including stage of immune depression, likely play a role in the occurrence of the HIV related PAH. It is possible that, the implication of HIV viral proteins be related with high level HIV-RNA, because detectable plasma HIV-RNA is associated with the risk to develop PAH, as demonstrated in a cross-sectional study [11, 46]. This can also explain why the prevalence of HIV-related PAH is high in Africa compared to developed countries, because HIV-infected patients in Africa have more HIV infection control failure compared to those in developed countries. The occurrence of pulmonary arterial hypertension is independent of the CD4 cell count, but it appears to be related to the duration of HIV infection [47].

### Role of genetic predisposition

Not all HIV infected develop PAH. Genetic predisposition plays a key role in the development of PAH among HIV infected people. Some major histocompatibility complex alleles have been incriminated by their high prevalence among HIV infected people with PAH, particularly HDL-DR6 and HLA-DR5 [48, 49].

It appears that the mechanism mainly specific to HIV-related PAH is due to viral proteins. Other mechanisms of HIV-related PAH are associated to either an increase in genetic predisposition or to an increase in the serum level of cytokines involved in the PAH.

## Histopathology

Histologically, HIV related PAH has the same characteristics with other types PAH [50]. There are three histopathological presentation including primary pulmonary arteriopathy (plexiform arteriopathy, thrombotic arteriopathy, isolated medial hypertrophy, and medial hypertrophy with intimal fibrosis), pulmonary veno-occlusive disease, and pulmonary capillary hemangiomatosis [51]. Concerning the importance of form, the most common histological form of HIV related PAH is the plexogenic pulmonary arteriopathy followed by thrombotic pulmonary arteriopathy, pulmonary medial hypertrophy with intimal fibrosis, and pulmonary veno-occlusive disease [50].

## Role of antiretroviral therapy in the treatment of HIV related pulmonary hypertension

There is no cure for pulmonary arterial hypertension [47]. It is essential to note that the treatment of HIV-related PAH is similar to the treatment of other forms of PAH. Several medications may be adjunctively (non-specific therapies) used in the management of PAH depending on the clinical presentation of PAH. These include: (1) anticoagulants because PAH patients are at risk for small intrapulmonary thrombus due to prothrombotic state, sedentary lifestyle, and cardiac dilation [22, 52]; (2) supplemental oxygen because patients with PAH often develop hypoxemia which can further exacerbate pulmonary arterial vasoconstriction [22, 52]; (3) diuretic agents which can be used in the case of volume overloaded; Digoxin in the case right sided heart failure [22, 52]; (4) Calcium channel blocker among PAH patients who develop decrease in mPAP between 10 mmHg and 40 mmHg. This reduction in mPAP must be accompanied by improved or at least unchanged cardiac output and unchanged or minimally reduced systemic blood pressure [22, 52, 53].

Several medicines are available in the specific management of PAH, the same both for non HIV-related PAH and HIV-related PAH, such as endothelin receptor antagonists, prostaglandin analogs, and phosphodiesterase 5 inhibitors [52].

There have been no large prospective studies of the effects of antiretroviral treatment on HIV related PAH. Most of the studies demonstrated the beneficial effect of the use of highly active antiretroviral therapy (HAART) among HIV infected patients with PAH on their outcome. As demonstrated in a cross sectional study, detectable plasma HIV-RNA was associated with the risk to develop PAH [11]. This suggests that the use of HAART, whose aim is to prevent the multiplication of HIV and thus make undetectable viral load; could reduce the risk of developing PAH in patients infected with HIV. In a prospective cohort study [54], highly active antiretroviral therapy (HAART) improved pulmonary artery pressure in HIV/AIDS patients if instituted at early stages (WHO classes I and II). However, at more advanced stages of pulmonary artery hypertension, it does not have any significant effect on reduction of the same. In the view of these results, early detection of PAH in HIV/AIDS patients is essential and prompt institution of HAART should be considered in them even when those patients do not fulfill the conventional criteria for initiation of this treatment. Further studies was needed to investigate these possibility. HAART should not be used as a sole therapy for HIV-related PAH, and the initiation of PAH-specific therapy is of paramount importance. HAART only ameliorate the outcome of patients with HIV-related PAH. In a cross-sectional study including 400 HIV infected patients, a current use of Tenofovir was associated with lower PAH prevalence [55]. Several other studies also show the beneficial effect of the use of HAART in the treatment of PAH [56, 57]. HAART also reduce mortality [58] and cardiac involvement [59] among HIV infected patients with PAH. Therefore, it is necessary that all patients with HIV-related PAH start HAART regardless HIV disease clinical stage and CD4 count. However, it is necessary to conduct large prospective studies to better estimate the effect of HAART on PAH.

## Conclusions

There is a high prevalence of PAH in patients infected with HIV in Africa, although there are very few studies, hence the need for further studies including research also risk factors. This prevalence is very high compared to that in the developed countries and compared with that in the general population. The pathophysiologic mechanism responsible for PAH in subjects infected with HIV remains to be explored. One notes nevertheless the involvement of HIV viral proteins, cytokines and genetic predisposition in the pathophysiologic mechanism. Although the pathophysiological mechanism of HIV-related PAH is different from other PAH, histological presentation is the same. The beneficial role of antiretroviral drugs in the treatment of PAH associated with HIV is not yet established, which requires large prospective studies to clarify.

## Abbreviations

BMPR2: bone morphogenic protein receptor 2
Gp120: glycoprotein 120
HAART: highly active antiretroviral therapy
HIV: human immunodeficiency virus
Nef: negative factor
PAH: pulmonary arterial hypertension
PH: pulmonary hypertension
SIV: simian immunodeficiency virus
Tat: transcription proteins
